# NUAK Kinases: Brain–Ovary Axis

**DOI:** 10.3390/cells10102760

**Published:** 2021-10-15

**Authors:** Ester Molina, Linda Hong, Ilana Chefetz

**Affiliations:** 1 The Hormel Institute, University of Minnesota, Austin, MN 55912, USA; molin396@umn.edu; 2 School of Medicine, Loma Linda University, Loma Linda, CA 92350, USA; Lihong@llu.edu; 3 Masonic Cancer Center, Minneapolis, MN 55455, USA; 4 Stem Cell Institute, Minneapolis, MN 55455, USA; 5 Department of Obstetrics, Gynecology and Women’s Health, University of Minnesota, Minneapolis, MN 55455, USA

**Keywords:** NUAK1, NUAK2, AMPK-related kinases, ovary, brain

## Abstract

Liver kinase B (LKB1) and adenosine monophosphate (AMP)-activated protein kinase (AMPK) are two major kinases that regulate cellular metabolism by acting as adenosine triphosphate (ATP) sensors. During starvation conditions, LKB1 and AMPK activate different downstream pathways to increase ATP production, while decreasing ATP consumption, which abrogates cellular proliferation and cell death. Initially, LKB1 was considered to be a tumor suppressor due to its loss of expression in various tumor types. Additional studies revealed amplifications in LKB1 and AMPK kinases in several cancers, suggesting a role in tumor progression. The AMPK-related proteins were described almost 20 years ago as a group of key kinases involved in the regulation of cellular metabolism. As LKB1-downstream targets, AMPK-related proteins were also initially considered to function as tumor suppressors. However, further research demonstrated that AMPK-related kinases play a major role not only in cellular physiology but also in tumor development. Furthermore, aside from their role as regulators of metabolism, additional functions have been described for these proteins, including roles in the cell cycle, cell migration, and cell death. In this review, we aim to highlight the major role of AMPK-related proteins beyond their functions in cellular metabolism, focusing on cancer progression based on their role in cell migration, invasion, and cell survival. Additionally, we describe two main AMPK-related kinases, Novel (nua) kinase family 1 (NUAK1) and 2 (NUAK2), which have been understudied, but play a major role in cellular physiology and tumor development.

## 1. Introduction

Metabolism comprises the processes responsible for supplying energy to cells to perform their various functions, including proliferation and survival. The main molecule that provides this energy is adenosine triphosphate (ATP). Thus, ATP production and consumption are important processes that are under tight regulation in the maintenance of normal cellular homeostasis.

The main cellular sources of ATP are produced through glycolysis and the tricarboxylic acid (TCA) cycle. Glycolysis is a central metabolic pathway, which converts monosaccharides, commonly glucose, into pyruvate [1]. In the presence of oxygen, pyruvate enters the TCA cycle to be converted to high-energy molecules, such as ATP or nicotinamide adenine dinucleotide hydrogen (NADH), which is utilized as a co-factor. Additionally, intermediates produced during the TCA cycle are utilized as precursors for the synthesis of cellular elements, such as lipids, proteins, and nucleic acids.

In the absence of oxygen, glucose is converted into lactate generating less ATP than that produced by the TCA cycle but is critical for the support of cellular survival in hypoxic conditions. Paradoxically, rapidly proliferating cancer cells preferentially use the glycolytic anaerobic pathway producing lactate even in the presence of oxygen. This process is commonly known as the Warburg effect and it has long been identified as a hallmark of cancer cells [2].

Furthermore, under stressful cellular conditions, such as lack of nutrients or oxygen, cells produce high levels of reactive oxygen species (ROS) that induce damage to lipids, proteins, and nucleic acids, eventually triggering cell death. Therefore, various cellular defense mechanisms are utilized to counteract these highly reactive molecules, including an increase in antioxidant levels, removal of defective proteins by the ubiquitin-proteasome system, and recycling damaged organelles through autophagy [3,4]. Consequently, many types of cells, including differentiated or highly proliferative normal cells (e.g., skin cells, mucous membranes, or hematopoietic cells) and tumor cells rely on these defense mechanisms to survive under stressful conditions.

One of the main features of tumor cells is their ability to adapt metabolic pathways to nutrient-deprived conditions or hypoxia. In order to survive under these stressful conditions, the production of ROS and the requirement of ATP are the two main events that tumor cells attempt to bypass.

### 1.1. Liver Kinase B (LKB) 1, Adenosine Monophosphate (AMP)-Activated Protein Kinase (AMPK) and AMPK-Related Kinases

The liver kinase B1 (LKB1) protein, is a serine (Ser or S) threonine (Thr or T) kinase that was originally described as the product of the primary gene that is mutated in the autosomal dominant human disorder referred to as Peutz–Jeghers syndrome (PJS) [5]. Although LKB1 was initially characterized as a tumor suppressor, further research revealed that LKB1 can act both as a tumor suppressor and oncogene because of its widespread activity in various tissues and its multiple downstream targets [6,7].

LKB1 is a key activator of adenosine monophosphate (AMP)-activated protein kinase (AMPK), which acts as the main sensor of the balance of ATP, adenosine diphosphate (ADP), and monophosphate (AMP). In order to maintain cellular energy requirements, AMPK can detect ATP depletion and consequently increases ADP or AMP levels. The allosteric binding of ADP and AMP to AMPK promotes AMPK activation and subsequent downstream target activation in order to increase ATP production [8,9].

LKB1-AMPK downstream targets were described in human tissues in 2004 as 13 novel rare kinases [10]. This group of kinases comprises Novel (nua) kinase family 1 (NUAK1), NUAK2, salt inducible kinase 1 (SIK1), QIK (also known as SIK2), QSK (also known as SIK3), as well as maternal embryonic leucine zipper kinase (MELK). These kinases are all activated by LKB1 on a highly conserved phosphorylation site. LKB1 also regulates microtubule affinity regulating kinases 1–4 (MARKs 1–4) and brain selective kinases 1 and 2 (BRSK1 and 2). Collectively, the above proteins are known as the AMPK-related family of proteins [11].

Besides their role in metabolism, AMPK-related kinases mediate the cell cycle, cell migration and polarization, and cell death. Many of these proteins, including NUAKs [12] and BRSKs, were initially identified and functionally characterized in murine and rat brains [13,14,15]. Furthermore, the SIK1 protein was discovered in the rat pheochromocytoma cell line (PC12), as part of an experiment that aimed to identify proteins activated as a result of neuronal depolarization [16]. Although the function of AMPK-related proteins in the brain has been extensively studied, their role in cancer has just begun to be described. For example, the SIK proteins have been recently described as downregulated in non-small cell lung cancer (NSCLC) [17] or activated in mouse models of pancreatic cancer [18,19]. The MARK proteins directly regulate microtubule organization and their function is associated with cellular polarization and migration [20,21]. Finally, MELK has been described as an important protein in oocyte maturation [22].

### 1.2. LKB1, AMPK, and AMPK-Related Proteins in Cancer

The role of AMPK in maintaining the ATP: ADP/AMP balance is also associated with tumor progression. This is because their main function is to balance ATP consumption versus ATP production in order to maintain the ATP levels required for proper cellular functions [8]. Additionally, AMPK downstream target kinases regulate ROS production [23,24]. Thus, AMPK proteins can confer the ability of tumor cells to grow and divide rapidly, while minimizing undesirable side effects, such as DNA damage, cell detachment, or cell death [23,24,25].

**Figure 1:**
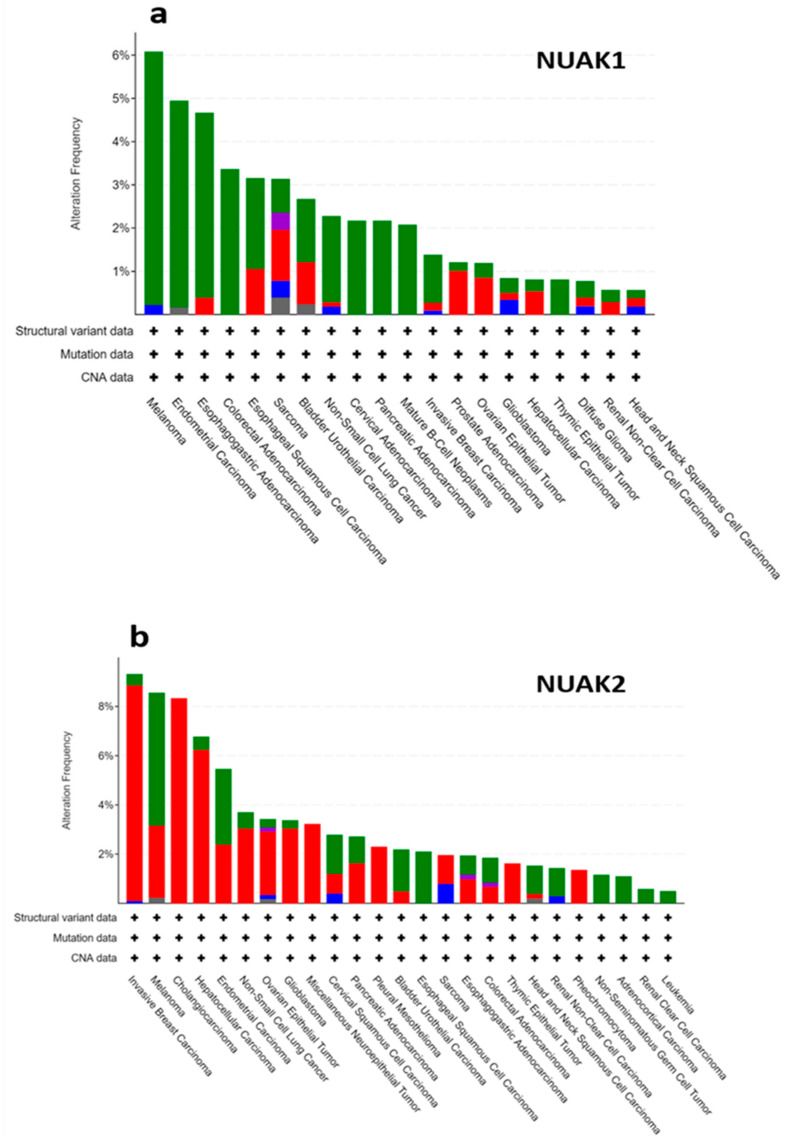
NUAK 1 and 2 gene alterations in the TCGA database. The graphs represent the percentage of alterations encountered in NUAK1 (**a**) and NUAK2 (**b**) from various patient cancer tissue samples, analyzed using the cBioportal database. NUAK1 is frequently mutated in many different cancer types. Higher NUAK1 mutation frequency has been found in colorectal cancer, endometrial cancer, and melanoma. Whereas NUAK2 is found broadly amplified in different cancer types with higher frequency in breast cancer and melanoma. Similar to NUAK1, NUAK2 has been also found altered in reproductive cancers as ovarian and prostate cancer, glioblastoma, and colorectal cancer.

Therefore, AMPK and AMPK-related proteins have not surprisingly been found to be deregulated, and mainly amplified, in various cancers [26]. Indeed, analysis of the cancer genome atlas (TCGA) database revealed NUAK1 and NUAK2 deregulation in lung, breast, liver, ovarian, and melanoma tumors (Figure 1a,b). In addition to the TCGA data suggesting the relevance of AMPK-related proteins to the development of various cancers, several experimental studies explored their potential role in tumor initiation and progression. For example, NUAK1 and NUAK2 downregulation has been linked to reduced motility and invasiveness of renal and breast cancer cell lines [27], whereas overexpression of NUAK2 in HepG2 human hepatocytes increases survival following glucose starvation [28,29]. More recently, NUAK1 overexpression was reported to be associated with poor prognosis in a murine model of colorectal cancer [30].

Despite recently accumulated evidence, only limited data are available regarding NUAK1 and NUAK2 and their role in cellular physiology, their potential downstream targets in various tissues, and how gene amplifications affect tumor development. In this review, we aim to unravel the role of NUAK1 and NUAK2 as members of the AMPK-related kinase family of proteins and to examine their various cellular functions, with a focus on the implications of altered expression and the potential subsequent modified activities in different cancer types.

### 1.3. The NUAK1 and NUAK2 Protein Kinases

The NUAK1 protein, originally identified as sucrose nonfermenting 1 (SNIF1)/AMPK-related protein, abbreviated (SNARK), was initially isolated from rat liver tissue as a part of a screening experiment aiming to identify proteins activated as a result of ultraviolet (UV)-damage [31]. Later on, NUAK1 was designated as an AMPK family member and named AMPK-related protein kinase 5 (ARK5). Initial observations revealed upregulated ARK5 expression upon glucose starvation in human hepatocarcinoma cell lines (HepG2) in a protein kinase B (PKB; or Akt)-dependent manner [12].

**Figure 2:**
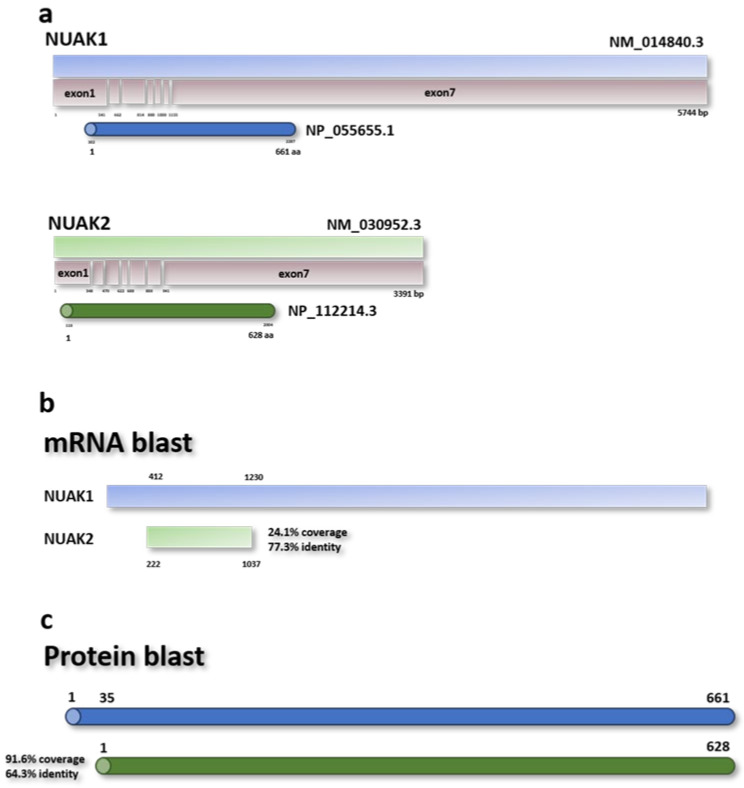
Scheme representation of the *NUAK1* and *NUAK2* genes. (**a**) The bars represent the length of the cDNA sequence, the exons encoded by the cDNA, and the coding DNA sequence that give rise to NUAK1 (above) and NUAK2 (below) proteins. NUAK1 is codified by 7 exons which expands for 5744 bp, whereas NUAK2 is codified by 3391 bp. However, both sequences translate into a similar size and sequence proteins. (**b**,**c**) Schematic representation of NUAK1 (blue) and NUAK2 (green) blast analysis of their mRNA (**b**) and protein (**c**) sequences. NUAK1 mRNA is longer compared with NUAK2, with a percentage of coverage around 24% with an identity (nucleotides in the same position) of 77%. NUAK1 and NUAK2 proteins have similar size but lower identity of around 64%.

On the other hand, NUAK2 was also reported in the same publication but was mistakenly named NUAK1 (SNARK) due to its high sequence similarity. These two proteins share 91.6% coverage and 64.3% identity (Figure 2c) [31]. Even though the role of NUAK2 is less studied compared to NUAK1, limited experimental studies characterize NUAK2 as an important kinase involved in cell motility and cell-cell detachment [29]. Finally, following additional confirmation and validation studies, NUAK proteins have been identified as a downstream target of LKB1 and AMPK, comprising a large family of 13 Ser/Thr AMPK-related kinases [10].

#### 1.3.1. NUAK Genes

**Figure 3:**
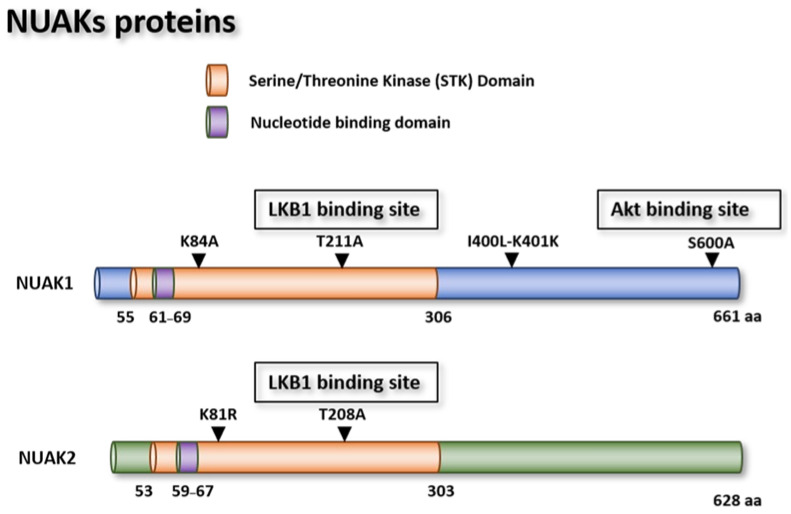
Schematic representation of the NUAK1 and NUAK2 proteins. The bars represent the different domains that characterize these proteins. Depicted are the different mutants described in the literature for NUAK1 (above) and NUAK2 (below) proteins. NUAK1 and NUAK2 proteins have two main domains. The STK domain is the kinase domain that characterizes this family of proteins. Inside the kinase domain contains the nucleotide-binding domain and the main residues that activates the kinase activity of this protein or the LKB1 binding site. NUAK1 also has the residue susceptible to be activated by Akt protein near the C-terminal part of the protein.

*NUAK1* is a gene encoding a 74 kiloDalton (kDa) protein located in the short arm of chromosome 12 (Figure 2a), comprising seven exons, which are translated into 661 amino acids (aa). As a Ser/Thr kinase, this protein possesses kinase and ATP binding domains, which are characteristic of this family of proteins. The binding residue for Akt-dependent activation of Ser600 is located in the C-terminal region of the protein (Figure 3) [12,32]. NUAK1 is ubiquitously expressed within various cell compartments, but its expression is more evident in the nucleus and cytoskeleton organelles.

##### NUAK1 Mutants

Different NUAK1 mutants have been described in the literature. For example, a lysine (K) 84 substitution by an alanine (A) mutant abolishes NUAK1 kinase activity and induces senescence in WI-38 normal lung cells. Cells transfected with this kinase mutant had an extended lifespan compared to their control counterparts, suggesting that this residue is necessary for NUAK1-induced senescence (Figure 3) [32,33]. In vitro kinase assay results indicated that a T211A mutation of NUAK1 impedes LKB1 phosphorylation and subsequent activation of NUAK1 [10]. Other in vitro experiments performed with human embryonic kidney (HEK) 293T cells revealed that mutations in residues isoleucine (I) 400K and leucine (L) 401K inhibit NUAK1′s interaction with the protein phosphatase 1 catalytic subunit beta (PPP1CB) protein and abolishes its interaction with the myosin cytoskeleton protein, interfering with cell adhesion [34]. Finally, the mutant S600A abrogates NUAK1 activation by Akt in HepG2 cells and in human diploid fibroblasts (Table 1) [29,32].

##### NUAK1 Tissue Expression

According to the National Center of Biotechnology Information database [37], in normal tissues, NUAK1 is highly expressed in the brain and skin, and to a lesser extent, in breast, heart, ovary, cervix, and lung tissues. NUAK1 levels are deregulated in various cancer tissues and cell lines, including brain cancer, melanoma, and different types of reproductive tissue-derived cancer cells, such as breast, ovarian, cervix, and prostate [37].

##### NUAK2 Gene

In humans, *NUAK2* is encoded in the short arm of chromosome 1. The *NUAK2* gene is comprised of seven exons that give rise to a 3.3 kilobase (Kb) single transcript variant (Figure 2a). The 69 kDa protein encoded by this gene has a kinase domain located in the N-terminal region similar to other Ser/Thr kinase protein family members. Although the NUAK2 transcript only presents less than 25% similarity with the NUAK1 transcript, both proteins possess more than 60% identity (Figure 2b). Finally, two different mutants have been described that affect NUAK2 cellular activity.

##### NUAK2 Mutants

The K82 arginine (R) mutant is an inactive NUAK2 mutant, due to its interference with the NUAK2 autophosphorylation site inhibiting its anti-apoptotic activity in the ACHN renal cell carcinoma cell line [27]. The K81 residue has a phosphotransferase activity in its enzymatic pocket. The K81 methionine (M) and T208A NUAK2 mutants have been described to allow hepatitis C virus (HCV) replication, whereas the NUAK2 wild type (WT) impairs HCV replication in Huh 7.5.1 hepatitis cells [36]. In addition, in vitro kinase studies revealed that the T208A NUAK2 mutant impairs LKB1 phosphorylation and activation [10] (Figure 3).

##### NUAK2 Tissue Expression

According to the NCBI database [37], NUAK2 is broadly expressed in different human tissues with higher levels found in the digestive tract, including the esophagus, small bowel, and colon, in addition to other organs, such as the kidney, spleen, and thyroid. However, TCGA database (Figure 1b) analysis exhibits alterations in NUAK2 expression, comprising mostly amplifications in reproductive organ tumors, such as breast, ovarian, and uterine, in addition to other tumor types, including melanoma, liver, and lung cancers [37].

#### 1.3.2. NUAK1 and NUAK2 in Cancer

Despite the limited number of studies that experimentally elucidate the role of NUAK1 and NUAK2 in cancer development, both kinases have been found amplified in different tumors. The role of NUAK1 in different tumor types has been recently reviewed in [38]. Here, we highlight the role of NUAK proteins in different pathways and their potential involvement in tumor initiation and propagation.

##### Apoptosis and Senescence

Initial reports regarding NUAK1 and NUAK2 function indicate a potential role in apoptosis resistance as assessed in MCF7 human breast cancer cells following starvation. Many signaling pathways contribute to the development of resistance to apoptosis, including nuclear factor kappa B (NFκB) and the phosphatidylinositol 3-kinase (PI3-K)/Akt pathways [35,39]. NUAK 1 and NUAK 2 have been characterized as downstream targets of the CD95/Fas cell death receptor (FAS), more commonly known as death receptor [35]. CD95-induced caspase-dependent apoptosis requires activation of the non-apoptotic NFκB and (PI3-K)/Akt pathways [40]. For instance, NUAK1 and NUAK2 expression is upregulated upon CD95 receptor stimulation and this effect was dependent on NFκB pathway activation. This suggests a pro-survival role for the NUAK proteins. CD95-induced apoptosis is severely impeded when NUAK1 and NUAK2 proteins were silenced in MCF7 breast cancer, human cervix carcinoma (HeLa) [35], and colorectal cancer cell lines [30,41].

Senescence is a cellular process induced by the exhaustion of cell replicative potential. When cells become senescent, the cell cycle is abrogated but the metabolic activity within the cell remains active. In addition, a senescent phenotype can be acquired by other means, such as cellular or oxidative stress or the activation of oncogenic signals [42]. In tumor cells, immortalization is commonly triggered by the deregulation of the retinoblastoma (Rb) and p53 pro-apoptotic pathways, which allows cancer cells to bypass senescence and extend their lifespan. NUAK1 expression has been shown to be upregulated in human diploid fibroblasts (HDFs) after they acquire a senescent phenotype [32]. Silencing NUAK1 expression with shRNAs confers the cells an extension of replicative potential. In contrast, NUAK1 overexpression accelerates the senescence phenotype in HDFs. Furthermore, NUAK1 senescent phenotype induction is LKB1-dependent and Akt-independent, as the S600A mutant does not induce senescence in these cells [32]. Furthermore, NUAK1 overexpression has been observed to induce aneuploidy through the downregulation of the large tumor suppressor homolog 1 (LATS1). LATS1 blocks cytokinesis inducing aneuploidy and consequently senescence in HDF [43]

Recently, a role for NUAK2 in cell cycle and proliferation mediated through the Yes-associated protein 1 (YAP) and WW-domain-containing transcription regulator 1 (WWTR1; also known as TAZ) pathway has been described [44]. YAP/TAZ proteins are negatively regulated by LATS1/2, through their retention in the cytosol and further degradation by the proteasome system [44]. When activated, YAP/TAZ proteins translocate into the nucleus and activate genetic programs involved in cell proliferation and survival [45]. In HuCCT-1 liver cancer cells, proliferation and survival are highly dependent on the YAP pathway. Studies performed in mouse models (e.g., YAP-inducible overexpression mouse models that cause hepatomegaly and eventually liver tumorigenesis) of liver cancer indicate that NUAK2 knockdown decreases cell proliferation in YAP-dependent liver tumors and HuCCT cells. Additionally, mice injected with Cre-inducible virus carrying shRNA to silence NUAK2 expression exhibit decreased levels of hepatocyte proliferation [46].

##### Migration and Invasiveness

Cancer cells have the capacity to migrate from primary tissue to invade other tissues or lymph nodes in a process called metastasis. Low oxygen conditions trigger enhanced migration and invasion capacity of tumor cells through the activation of the Akt pathway [47]. Indeed, NUAK1 protein has been extensively described as a mammalian target of rapamycin (mTOR) downstream target, which is activated upon conditions of glucose starvation. Additionally, NUAK2 silencing has been associated with mTOR downregulation in cellular models of melanoma [48]. Therefore, NUAK proteins could possess tumor growth supporting functions given that the mTOR pathway is a pro-survival pathway commonly downregulated in cancer [49]. For instance, the pancreatic cancer cell line, PANC1, is very resistant to glucose starvation. Glucose starvation induces cell cycle arrest and apoptosis that can be bypassed in cancer cells through the activation of the Akt pathway. NUAK1 overexpression in pancreatic and HepG2 cells increases resistance to glucose starvation in an Akt-dependent manner. Furthermore, these NUAK1-overexpressing cells form larger tumors with higher metastatic potential in BALB-c mice [28]. NUAK1 overexpression on the protein level has also been associated with poor prognosis and shorter overall survival in patients with late-stage nasopharyngeal [50], gastric [51], or non-small cell lung cancer (NSCLC) [52]. Likewise, NUAK2 silencing in different melanoma cell lines reduces the migration and invasion of these cells. NUAK2 depletion correlates with mTOR downregulation. Additionally, melanoma cancer cells with NUAK2 knockdown manifest reduced tumorigenicity in nude mice [48].

Studies performed in the mouse Kirsten rat sarcoma viral oncogene (KRAS) Cre-inducible model of colorectal cancer showed that NUAK1 was detected in spheroids released from tumor cells. Pharmacologic inhibition with highly specific NUAK1 inhibitors (HTH-01-015 or WZ4003) reduced the ability of these tumors to form spheroids with a tumor-initiating capacity [30].

One of the main biological processes involved in tumor metastasis is epithelial–mesenchymal transition (EMT). During EMT, epithelial cells detach from the surface of their surrounding tissue acquiring cellular transition that allows them to transform into a mesenchymal phenotype. The mesenchymal cells are able to migrate, invade neighboring tissues, and secrete extracellular matrix (ECM) components. In all tissues, the EMT process occurs naturally in order to renew a pool of mesenchymal cells or as a response to tissue injury, which is often associated with inflammation. In both cases, the EMT program is activated to renew certain cell populations and to maintain normal function. However, in tumor tissues, EMT can cooperate with active oncogenic pathways to allow tumor invasion and metastasis [53]. Additionally, some studies have linked EMT with chemoresistance in different tumors. For instance, doxorubicin is the most common treatment in hepatocellular carcinoma (HCC), and acquired chemoresistance to doxorubicin in HCC is associated with activation of the PI3-K/Akt and mitogen-activated protein kinase (MAPK) signaling pathways. Studies performed in HCC cell lines suggest the role of NUAK1 in the development of doxorubicin resistance is mediated through the induction of EMT [54]. Furthermore, hypoxia is known to induce EMT, subsequently allowing cells to survive anticancer treatments. Downregulation of NUAK1 expression reverses doxorubicin chemoresistance even under hypoxic conditions [46].

NUAK2 is also involved in HCC disease through the activation of hepatitis C virus (HCV) replicative potential mediated by the transforming growth factor β (TGF-β) signaling pathway. TGF-β signaling is an important pro-fibrogenic factor in the liver and NUAK2 silencing in Huh7.5.1 hepatocellular carcinoma cells reduce TGF-β signaling. Consequently, NUAK2 facilitates HCV replicative potential and allows pro-fibrogenic TGF-β signaling, which accelerates hepatic fibrosis progression and eventually causes hepatocellular carcinoma [36].

Similar observations have been made in breast cancer cell lines. Overexpression of NUAK1 in MDA-MB-231 breast cancer cells increases the migration and invasion potential of these cells in an Akt-dependent manner, suggesting a role for NUAK1 in EMT and metastasis. Indeed, NUAK1-overexpressing MDA-MB-231 cells were able to induce pulmonary metastasis in nude mice to a higher extent than breast cancer control cells [55].

#### 1.3.3. The NUAK1 and NUAK2 Regulatory Network

**Figure 4:**
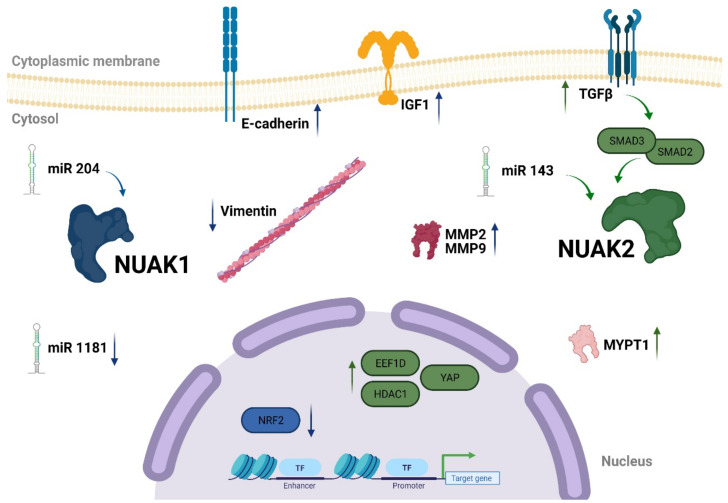
Schematic representation of NUAK1 and NUAK2 upstream and downstream targets. NUAK1/2 downstream targets are indicated with blue arrows (NUAK1) and green arrows (NUAK2). The direction of the arrow indicates whether the regulation of NUAK1/2 is direct (up arrow) or inverse (down arrow). Depicted in the cytoplasmic membrane are different protein receptors. Cytoskeleton proteins as well as cytoplasmic proteins and micro RNAs are represented in the cytosol. Proteins with square icons represent transcription factors. NUAK1 downstream targets involve different members of adhesion and cytoskeleton proteins such as vimentin and E-cadherin which share a similar role in invasion and metastasis. NUAK2 regulates different transcription factors related to pro-inflammatory cytokines which in turn activate the TGF-β signaling pathway ending a positive feedback loop. Conversely, NUAK1 protein negatively regulates TGF-β downstream signaling targets. Figure created in Biorender.com.

As indicated earlier, the role of NUAK1 and NUAK2 in cancer has been observed across various tumor types. In this manner, determining the regulatory network that involves NUAK1 and NUAK2 in tumor development is important (Figure 4).

NUAK1 overexpression correlates with nuclear factor erythroid 2-related factor 2 (NRF2) inhibition in different cancer cell lines. NRF2 is an emergency response element to oxidative stress [30]. Depletion of NUAK1 in bone cancer cell lines (U2OS) and different colorectal cancer cell lines triggers sensitization to oxidative stress and consequently cell death. Notably, NUAK1 inhibition induces NUAK2 overexpression suggesting that the role of NUAK1 in ROS-induced accumulation of NRF2 is partially shared with NUAK2. Indeed, depletion of NUAK2 in colorectal cell lines suppressed peroxide-induced NRF2 upregulated expression in the nucleus.

NUAK1-downregulation increases E-cadherin expression and decreases vimentin in hepatocellular carcinoma (HCC) cells [54] and gastric cancer (GC) tissue [56]. Moreover, NUAK1 silencing reduces the invasive capacity of HCC and GC cells in vitro and reduces tumor formation in vivo. Similar results have been observed in breast cancer cell lines. Akt-dependent NUAK1 activation increases breast cancer metastatic potential in MDA-MB-231 highly metastatic (MDA-MB-213HM) breast cancer cells [55]. Accordingly, the less invasive parental cell line, MDA-MB-213, exhibits decreased NUAK1 expression. Additionally, MDA-MB-213HM cells with NUAK1 knockout generate less invasive tumors in nude mice. Furthermore, NUAK1 overexpression correlates with matrix metalloproteinase (MMP) protein activation (MMP-2 and MMP-9), which is commonly associated with invasion and metastasis, suggesting that *MMP-2* and *MMP-9* are NUAK1-direct target genes. In addition, NUAK1 increases invasion in tumor cells mediated through MMP-2 and MMP-9 proteins and has been observed in NSCLC cells [52]. Finally, NUAK1 regulates insulin growth factor 1 (IGF1) through the Akt pathway in multiple myeloma (MM) cells. Akt-dependent NUAK1 activation increases the invasiveness of MM cells in in vitro models [41].

Micro (mi)RNAs are short (approximately 21–22 nucleotides) RNA molecules that affect the regulation of their target genes post-transcriptionally. MiRNAs bind to messenger RNA (mRNA) by sequence homology and block their translation. The miRNAs have great potential as prognostic and predictive biomarkers, as well as potent therapeutic tools through their regulation of oncogenes or tumor suppressor gene expression [57,58]. A few miRNAs interacting with the NUAK1 network in different tumor types have been described. For example, miR-204 directly regulates NUAK 1 expression and increases migration and metastasis in different NSCLC cell lines and mouse models. Stable NUAK1 transfection in NSCLC cells increases their chemotaxis and wound healing abilities; whereas, NUAK1 silencing in cell lines with high NUAK1 expression reduces their migration and invasion capabilities [52]. MiR-204 downregulates NUAK1 expression and consequently decreases cell invasion and metastasis of NSCLC cell lines. Indeed, miR-204 is downregulated in NSCLC tumor cells and patients’ tissue samples. These findings indicate that NUAK1 increases NSCLC invasion and metastasis.

NUAK1 upregulation has been reported in ovarian cancer and silencing of NUAK1 results in reduced cell migration in HEY ovarian cancer cells. miR-1181 has been described as a suppressor of the mesenchymal stem cell phenotype, inducing epithelial transition. NUAK1 downregulates miR-1181 expression, which leads to upregulation in EMT markers that cause increased invasion and migration. Conversely, NUAK1 silencing exhibits the epithelial phenotype reducing the invasion and wound healing ability of these cells [59]. It has been hypothesized that many oncogenes exert their function due to the downregulation of miRNA that controls their expression. In the A172 glioblastoma cell line, transient transfection of miRNA-143 downregulated NUAK2 expression which in turn reduced the proliferation, migration, and invasion potential of these cell lines [60].

Proteomic analysis performed in hepatocytes and 3T3 L1 CAR adipocytes cell lines demonstrated that NUAK2 regulates eukaryotic elongation factor 1δ (EEF1D) and histone deacetylase 1 (HDAC1). Cells with EEF1D knockout revealed the increased release of proinflammatory cytokines. In contrast, HDAC1 knockout did not mimic NUAK2 downregulation. Importantly, downregulation of HDAC1 and 2 simultaneously recapitulated the NUAK2 knockout phenotype. These findings demonstrate that NUAK2 regulates proinflammatory pathways through the upregulation of EEF1D and HDAC1 in hepatocytes and adipocyte cells [61]. Moreover, NUAK2 has been shown to be upregulated by SMAD2 and LKB1 in HCC Huh7.5.1 cells, activating the TGF-β signaling pathway [36].

In liver cancer HuCCT-1 cell line, YAP binds to the NUAK2 promoter upregulating its expression. Indeed, NUAK2 exhibits high expression in a large cohort of human liver tissue samples. NUAK2 phosphorylates protein phosphatase 1 regulatory subunit 12A (MYPT-1) on Ser445, which subsequently triggers the activation of actin fibers and creates actomyosin tension. As a next step, actomyosin tension activates YAP signaling ending this positive feedback loop [46]. A case report of a family with three consecutive fetuses born with anencephaly identified a recessive deletion in the NUAK2 gene, indicating a possible genetic cause. Indeed, experiments performed in iPSC-derived neural cells and cerebral organoids manifested that NUAK2 depletion decreases YAP/TAZ signaling pathway [62].

Recent reports support the role of NUAK proteins in TGF-β signaling, indicating that NUAK proteins play an opposite role in its activation. Indeed, it has been demonstrated that both kinases are upregulated upon TGF-β activation. However, while NUAK2 further enhances the activation of this pathway by stabilizing SMAD3/SMAD2 proteins, NUAK1 transcriptionally represses the activation of TGF-β downstream targets. Silencing of NUAK2 protein with siRNAs in a keratinocyte cell line (HaCaT) inhibits the activation of TGF-β downstream targets, whereas NUAK1 downregulation increases the activation of the pathway [63]. Additionally, similar results were observed in TGF-β-dependent contractility of myofibroblast, in which NUAK1 depletion induces myofibroblast contractility while NUAK2 downregulation abrogates this function [63]. In the C3H101/2 mouse embryonic fibroblast cell line, NUAK1 silencing prevented fibroblast differentiation towards myofibroblast. Conversely, NUAK2 upregulation induces MyoD or myogenin protein activation mediated by SMAD3, confirming the opposite role of NUAK proteins in the TGF-β-dependent signaling pathway [64]. Recently, NUAKs involvement in the TGF-β signaling pathway has been extensively reviewed [65].

#### 1.3.4. NUAK1 and NUAK2 in Ovary and Brain

Brain metastasis that originates from ovarian cancer is very rare; however, some clinical studies have linked brain cancer development to ovarian cancer [66]. At the molecular level, abnormal levels of brain-derived neurotrophic factor (BDNF) have been associated with depression with a higher incidence in women than men. Conversely, ovarian hormones have been described to affect BDNF levels at the mRNA and protein levels [67]. Overall, these data suggest a possible connection between the brain and ovarian signaling pathways. Results of a major miRNA screening of different patient samples of high-grade serous ovarian cancer (HGSOC) stratified patients into low, intermediate, and high-risk groups depending on total survival following diagnosis. The analysis revealed a major role of two main pathways in ovarian cancer: (a) cell motility and migration and (b) apoptosis, pro-survival, and cell death. Patients with poor survival prognosis or in the high-risk group revealed greater expression of EMT markers that also correlated with high chemotherapy resistance after surgery. Additionally, these patients presented with increased activity of the TGF-β, MAPK, PI3-K/Akt, or p53 pathways, in accordance with these pathways being usually deregulated in cancer. Interestingly, two other pathways emerged as key pathways in ovarian cancer development, the progesterone-mediated oocyte maturation pathway and the neurotrophin signaling pathway [68].

In mammals, the neurotrophin signaling network is mainly composed of nerve growth factor (NGF), BDNF, neurotrophin 3 (NT3), and NT4/5. NT proteins are required for survival and differentiation into brain neuronal and peripheral nervous system cells. Although NTs were previously suggested as only necessary for the development of the central nervous system (CNS), recent findings revealed different functions in the cardiovascular, immune, endocrine, and reproductive systems [69]. Experimental procedures performed in ovaries from feto-neonatal rodents revealed that NTs are abundantly expressed in somatic cells, including granulosa and mesenchymal cells [70,71].

These findings indicate the possibility that ovarian cancer and brain cancer share common pathways that, if deregulated, induce tumorigenesis in these apparently unrelated cancer types. Indeed, metastatic progression from ovarian cancer to brain cancer is extremely rare [72]. However, as previously indicated, a key pathway in neural development, such as the NTs signaling network, is also fundamental during oocyte development.

**Figure 5:**
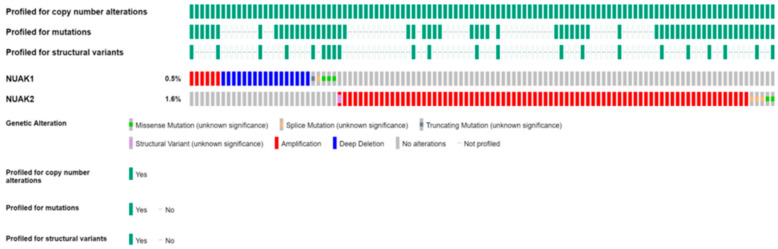
NUAK1 and NUAK2 alterations in different brain cancer tumors. cBioportal database data of NUAK1 and NUAK2 alterations in different brain cancer tumors from 19 different studies of human patient samples. From a total of 1102 patient samples, NUAK1 was altered in 0.6%. Alterations included amplifications, deletions, or mutations. In NUAK2, overall amplifications were detected in 1.7% of these patient samples.

Additionally, NUAK1 and NUAK2 are altered in both ovarian (Figure 1) and brain cancer (Figure 5). Although the frequency of alterations is low, it suggests a possible association between NUAKs expression and brain tumors. Indeed, the Protein Atlas database indicates that NUAK1 upregulated expression is correlated with bad prognosis in ovarian cancer [73,74] (Table 2) (Supplementary Figure S1A). Additionally, NUAK1 has been also shown to be upregulated in glioma patient samples. Indeed, statistical analysis for survival rate in glioma showed that NUAK1 upregulation is associated with poor survival, although it was not statistically significant (*p* = 0.051) (Supplementary Figure S1B). Intriguingly, curated data from glioma male patient samples exhibited a survival rate similar to those samples with low NUAK1 expression (Supplementary Figure S1D). On the contrary, curated data from glioma female patient samples revealed a significant correlation between NUAK1 overexpression and poor survival (Supplementary Figure S1C). In summary, these data indicate that NUAK1 upregulation is associated with bad prognosis in ovarian cancer patients and in female glioma patients, suggesting an interesting correlation between ovarian and brain cancers.

NUAK2 mRNA levels in ovarian cancer are not related to a lower survival rate (Table 2) (Supplementary Figure S2A), whereas high NUAK2 expression in glioma patient samples decreases the survival rate from 13% to 6% (Supplementary Figure S2B), indicating poor prognosis. However, there is no dependency on the gender of the patients in this case (Supplementary Figure S2C,D).

In addition to in silico data, NUAK2 expression was upregulated in an in vitro model of cultured cells from rat cerebellar granule neurons. These cells underwent apoptosis when potassium (K+) was increased in the culture media. This process was abrogated after the addition of BDNF or insulin growth factor (IGF) to culture media. NUAK2 was observed to be upregulated after K+ addition, leading to apoptosis in these cells [75].

LKB1 has been described to determine axon branching through their downstream targets, BRSK1 and BRSK2 [14,76]. Additionally, recent reports indicate NUAK1 and NUAK2 expression in the mouse brain during embryogenesis. However, NUAK2 expression disappears after birth whereas NUAK1 expression can still be detected in adults at both the mRNA and protein levels. Furthermore, NUAK1 knockout mice exhibit a reduction in axon branching to the same extent as LKB1 knockout mice, suggesting that LKB1 regulates axon branching through either BRSKs or NUAKs [77]. As mentioned before, NUAK2 deletion has been associated with anencephaly in humans. NUAK2 downregulates the YAP signaling pathway. It has been demonstrated that YAP/TAZ knockout mice failed to form a proper neural tube at embryonic stage 10.5 [78]. Additionally, NUAK2 has been found upregulated in different glioblastoma tumors in comparison with their normal counterparts [60].

Alzheimer’s disease (AD) is the most prevalent neurodegenerative disease and is characterized by the accumulation of β-amyloid plaques caused by Tau protein hyperphosphorylation [75]. An engineered human-medulloblastoma cell line expressing Tau-green fluorescent protein (GFP) chimeric protein indicated that NUAK1 downregulation decreases Tau levels. Furthermore, mice with a haploinsufficiency in NUAK1 revealed a decrease in Tau in the brain; whereas human tissues from AD patients revealed that Tau protein overexpression correlates with NUAK1 upregulation. In vitro kinase assay results confirm that NUAK1 stabilizes Tau by phosphorylation on Ser356 suggesting that NUAK1 is responsible for the development of AD by stabilization of the Tau protein [79].

On the other hand, NUAK1 has been demonstrated to play an important role in the development of ovarian cancer. Aside from the regulation of miR-1181 in ovarian cancer cells, NUAK1 overexpression has been shown to correlate with poor prognosis in ovarian cancer. NUAK1 downregulation with siRNA in OV90 ovarian cancer cells revealed that NUAK1 downregulation does not affect the chemoresistance of these cells to cisplatin or paclitaxel. In contrast, NUAK1-silencing influences cell migration and invasion as revealed in a wound-healing assay. Consequently, the NUAK1 correlation with poor prognosis in ovarian cancer might be partially due to increased migration and invasion [80]. Accordingly, an independent in vitro study demonstrated that NUAK1 downregulation reduces invasion of ovarian cancer cells. Indeed, NUAK1 downregulation in SKOV3 ovarian cancer cells reduced chemotaxis in an epidermal growth factor 1 (EGF-1)-induced experiment [81].

A recent study demonstrated that NUAK1 is also involved in spheroid formation in high-grade serous ovarian cancer cell lines (HGSOC). NUAK1 expression is downregulated in spheroids in comparison to adherent cells. Additionally, three different cell lines were assessed with different NUAK1 expression levels: Ovcar8 (high expression), and Ovcar3 and Heya8 (almost undetectable or low expression). Although the Ovcar8-NUAK1 knockout cell line lost cell adhesion, Ovcar3 and Heya8-NUAK1 exhibited increased cell adhesion capacity. These observations indicate that NUAK1 enhances epithelial ovarian cancer cell adhesion. Additionally, in vitro experiments demonstrated that fibronectin1 (FN1) protein level is correlated with NUAK1 expression, suggesting that NUAK1 regulates EMT transition in ovarian epithelial cancer cells through FN1 [82].

Finally, experimental procedures performed in two independent cohorts of ovarian cancer patient samples indicate that *NUAK1* is one of the most upregulated genes in patient samples with poor prognosis of the disease [83].

## 2. Conclusions

The AMPK-related family of proteins plays an important role in cellular metabolism. The LKB1 and AMPK pathways regulate cellular homeostasis and maintain the balance of ATP based on cellular energetic requirements. Although LKB1 was initially considered to be a tumor suppressor, further research has demonstrated that LKB1 and its downstream targets are amplified in different tumor types and their overexpression is often associated with poor prognosis.

NUAK1 and NUAK2 were previously described in rodent brain tissue and their expression has been subsequently confirmed in different human tissue samples, including those of reproductive organs. Additionally, NUAK1 and NUAK2 overexpression has been found in various tumor types and their upregulation is often correlated with an increased migration and invasion capacity. Furthermore, the NUAK1 and NUAK2 proteins regulate other essential pathways involved in cell physiology and tumorigenesis, such as cell cycle progression, metabolism, and cell death. Overall, evidence suggests that NUAK1 and NUAK2 play an important role in tumor development as protein kinases and could be appealing therapeutic targets.

Interestingly, brain and ovarian tissues have common signaling pathways associated with development and differentiation. Indeed, the protein atlas database [73,74,84] predicts many altered genes in common between reproductive tissues and brain cancer samples (https://www.proteinatlas.org). Initially, NUAK1 and NUAK2 were described in brain tissue, however, further research has demonstrated their role in cell migration and polarization, as well as invasive and metastatic processes in cancer cells. Furthermore, the importance of NUAK1 in ovarian cancer has been demonstrated in tumor development. NUAK1 overexpression correlates with poor prognosis in in vitro cell culture models and patients’ tissues. Clearly, these investigations point out the possibility of a major role of the NUAK1 and NUAK2 protein kinases in tumor development and support their potential role as novel therapeutic targets.

## Figures and Tables

**Table 1:** NUAK1 and NUAK2 mutations in different cell and animal models.

Gene	Mutant	Function	Model	Reference
**NUAK1**	S600A	Abrogates Akt activation	HepG2 (human liver cancer) Wi-38 (human diploid fibroblast, HDF)	[28,31]
	K84A	Abolishes NUAK1 kinase activity	Wi-38 (human diploid fibroblast, HDF) A549 (human lung cancer)	
	T211A	Abrogates LKB1 activation	In vitro kinase assay	[10]
	I400K L401K	Abrogates binding with myosin cytoskeleton proteins	HEK 293T (human embryonic kidney)	[34]
**NUAK2**	K82R	Dead kinase mutant	ACHN (renal carcinoma cell line)	[35]
	K81M T208A	Abrogates phosphotransferase activity of NUAK2 and LKB1 activation	Huh 7.5.1 (human hepatocyte derived cellular carcinoma) In vitro kinase assay	[10,36]

**Table 2:** Protein Atlas data from patient samples. Survival rate data analysis in ovarian cancer and glioma.

Gene	Cancer Type	Gender	Number of Samples	Gene Expression	Survival Rate	*p*-Value
NUAK1	Ovarian cancer	Total	373	High	24%	0.0041
				Low	38%	
	Glioma	Total	153	High	8%	0.051
				Low	10%	
		Female	54	High	7%	0.033
				Low	13%	
		Male	99	High	8%	0.28
				Low	9%	
NUAK2	Ovarian cancer	Total	373	High	37%	0.092
				Low	28%	
	Glioma	Total	153	High	6%	0.012
				Low	13%	
		Female	54	High	5%	0.12
				Low	18%	
		Male	99	High	6%	0.06
				Low	12%	

## Data Availability

https://www.cbioportal.org/ (accessed on 20 August 2021); https://www.proteinatlas.org/ (accessed on 20 August 2021).

## Supplementary Materials

The following are available online at https://www.mdpi.com/article/10.3390/cells10102760/s1, Figure S1: Kaplan-Meier plots survival rate in ovarian and glioma patients depending on NUAK1 expression. Figure S2: Kaplan-Meier plots survival rate in ovarian and glioma patients depending on NUAK2 expression.
