# Supplementary material for: NUAK Kinases: Brain–Ovary Axis
Source: Cells. 2021 Oct 15;10(10):2760. doi: 10.3390/cells10102760 (PMC8535158; doi:10.3390/cells10102760)
Supplement: Supplementary file 1 [file cells-10-02760-s001.zip › cells-1369296-supplementary.pdf]

# Supplementary figures

**Supplementary figure 1 (Figure S1)**. Kaplan-Meier plots survival rate in ovarian and glioma patients depending on NUAK1 expression.

*Supplementary Figure 1A*

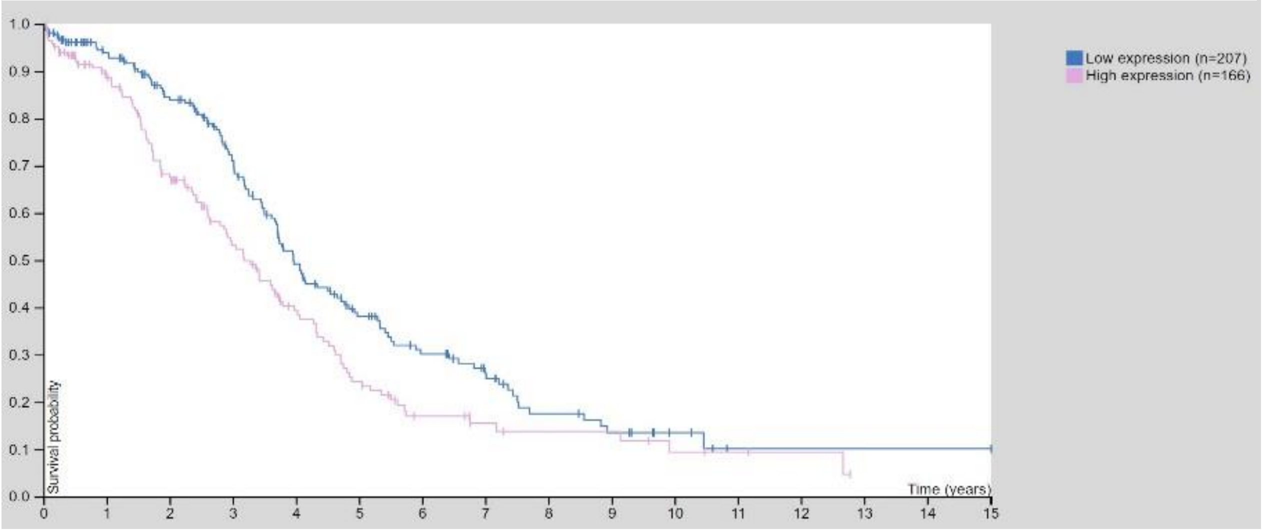


*Supplementary Figure 1B*

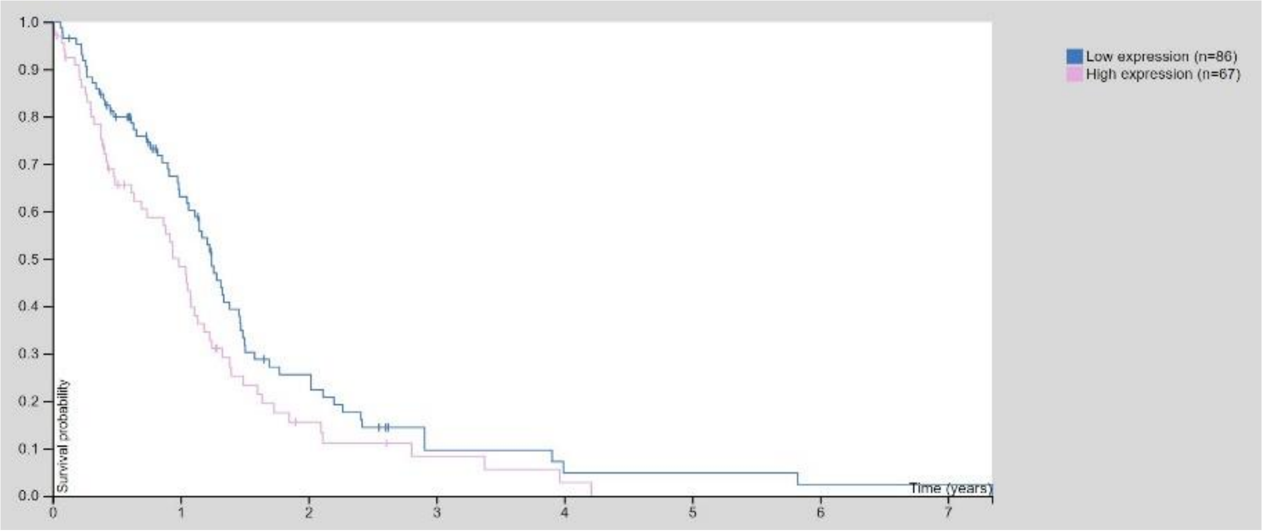

*Supplementary Figure 1C*

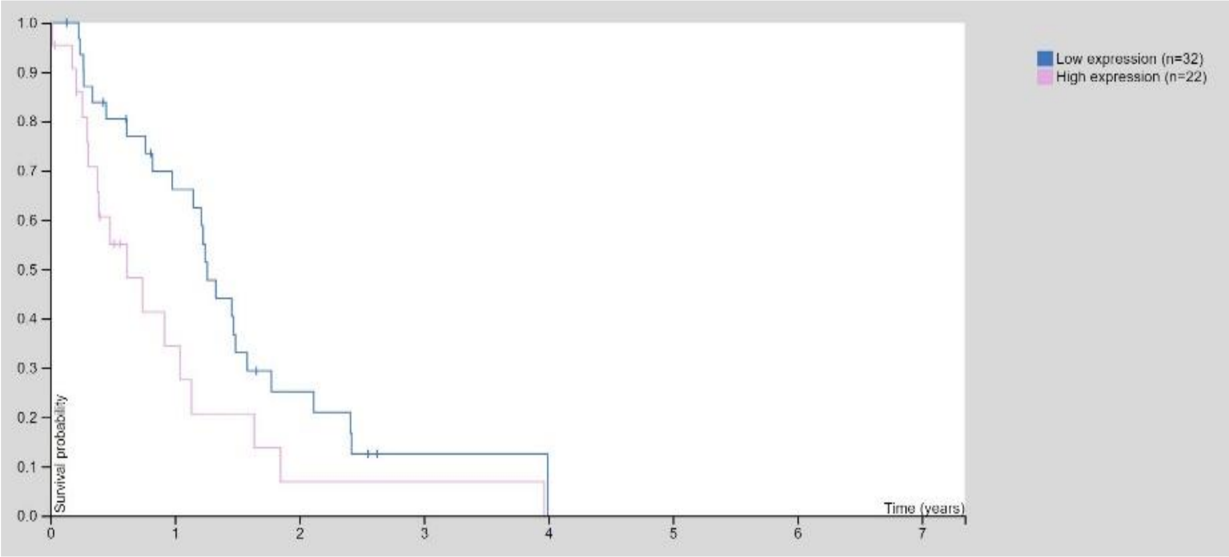


*Supplementary Figure 1D*

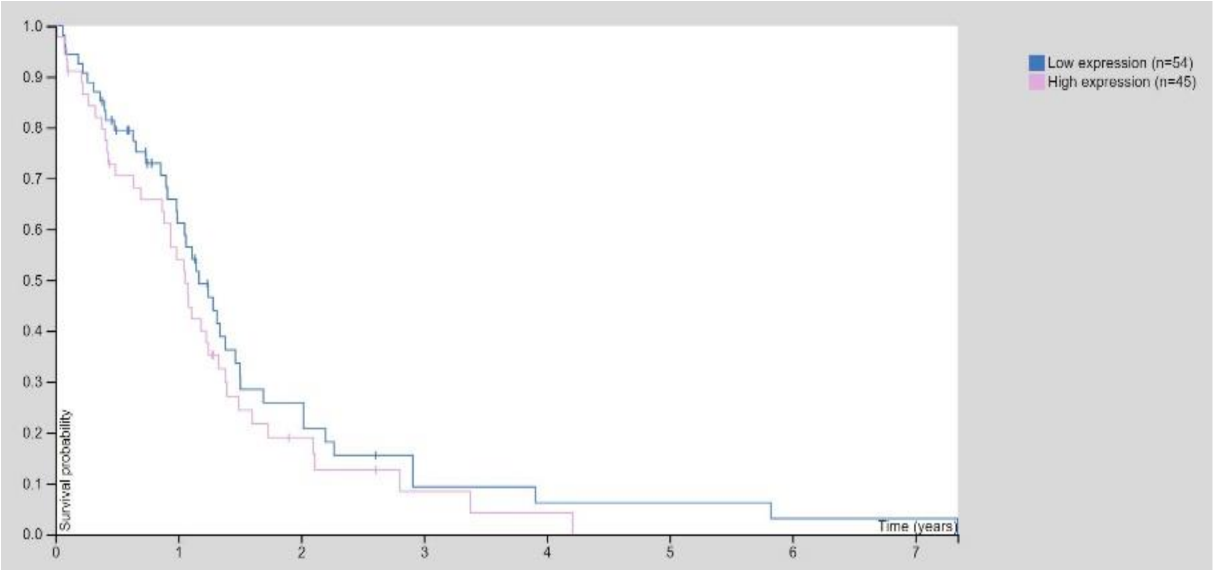


Kaplan-Meier plots survival rate in ovarian (a) and glioma (b,c&d) patients related to NUAK1 high expression (pink line) and low expression (blue line). NUAK1 high expression correlates with poor prognosis of the disease in ovarian cancer and in female glioma patient samples.

**Supplementary figure 2 (Figure S2).** Kaplan-Meier plots survival rate in ovarian and glioma patients depending on NUAK2 expression.

*Supplementary Figure 2A*

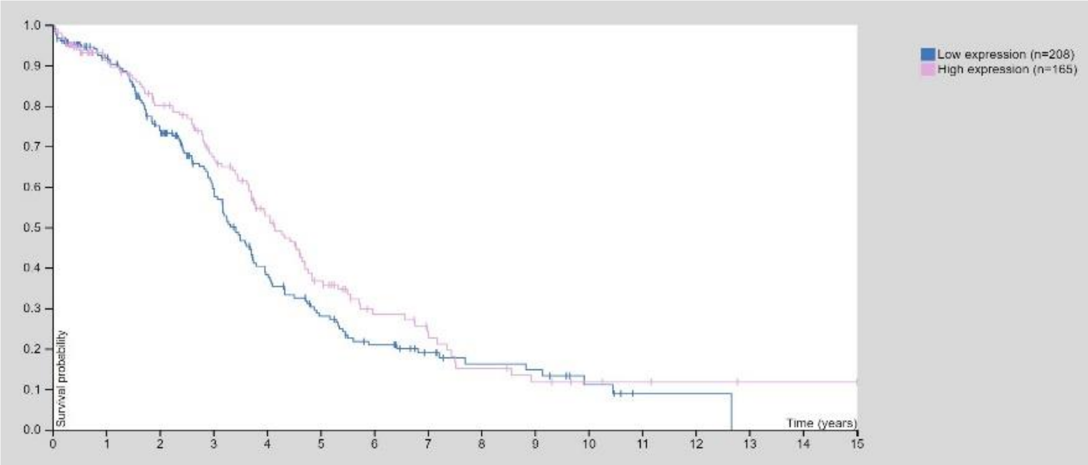


*Supplementary Figure 2B*

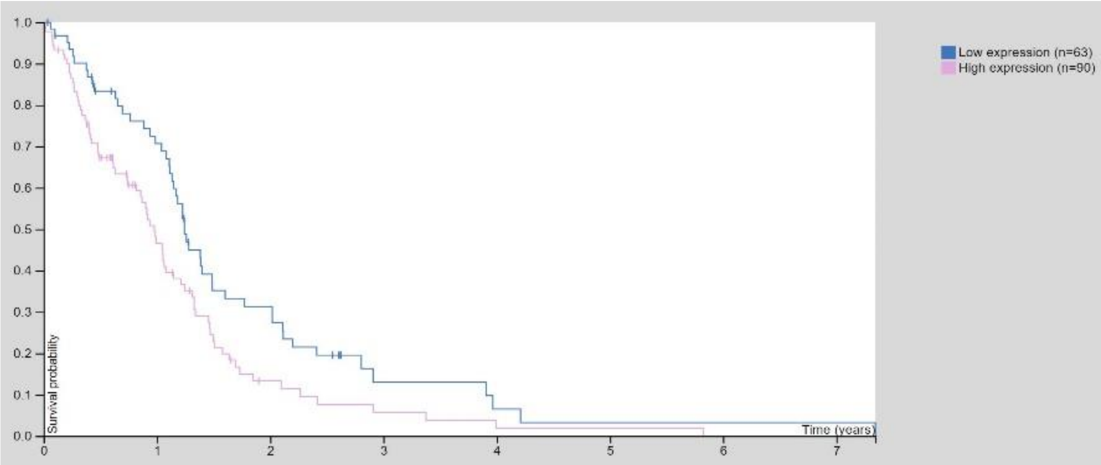

*Supplementary Figure 2C*

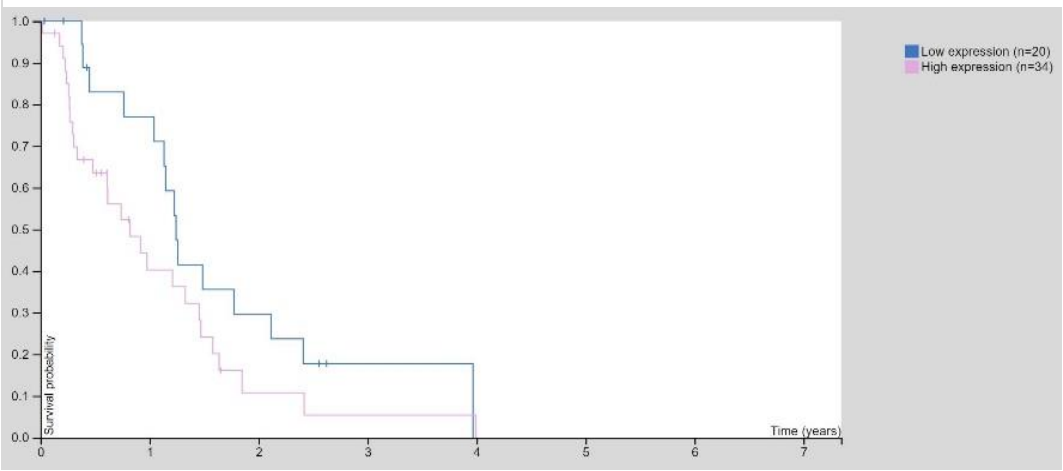


*Supplementary Figure 2D*

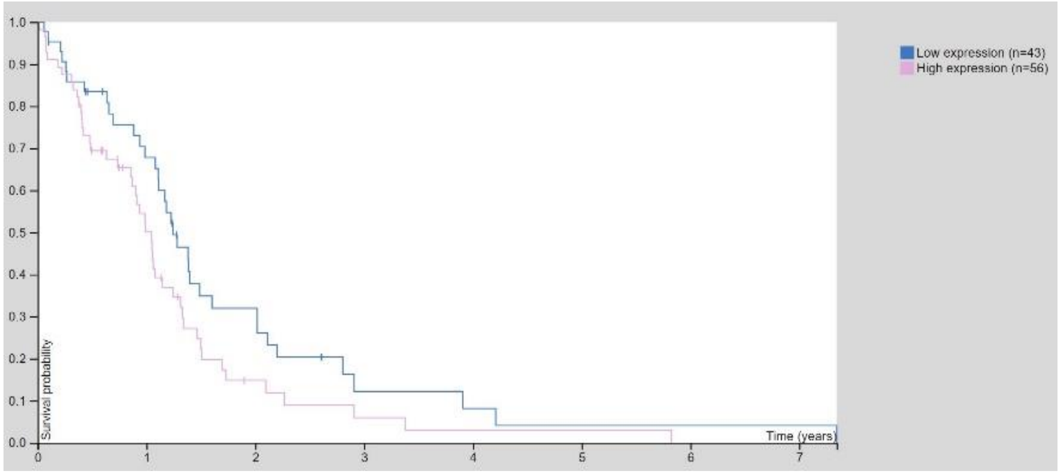


Kaplan-Meier plots survival rate in ovarian (a) and glioma (b,c&d) patients related to NUAK2 high expression (pink line) and low expression (blue line). NUAK2 high expression correlates with poor prognosis of the disease in glioma patient samples. However, NUAK2 high levels shows no changes between gender is glioma patient samples.
